# Psychological contract breach and job performance of new generation of employees: Considering the mediating effect of job burnout and the moderating effect of past breach experience

**DOI:** 10.3389/fpsyg.2022.985604

**Published:** 2022-09-09

**Authors:** Dongping Yu, Ke Yang, Xinsi Zhao, Yongsong Liu, Shanshan Wang, Maria Teresa D’Agostino, Giuseppe Russo

**Affiliations:** ^1^International Business School, Yunnan University of Finance and Economics, Kunming, China; ^2^Business School, Yunnan University of Finance and Economics, Kunming, China; ^3^International Languages and Cultures School, Yunnan University of Finance and Economics, Kunming, China; ^4^College of Innovative Business and Accountancy, Dhurakij Pundit University, Bangkok, Thailand; ^5^Department of Economics and Law, University of Cassino, Cassino, Italy

**Keywords:** new generation of employees, psychological contract breach, job burnout, job performance, past breach experience

## Abstract

With the intensification of COVID-19 epidemic, it becomes prominent to discuss the issue about the influence of psychological contract breach on job performance of new generation of employees. Based on social exchange theory, fairness theory, and conservation of resource theory, this study constructed a relationship model between psychological contract breach and job performance of new generation of employees with considering the mediating effect of job burnout and the moderating effect of past breach experience. Our hypotheses were tested using data from 235 respondents working in Yunnan Province, China. The results were as follows: first, psychological contract breach had a significant negative effect on job performance of new generation of employees, whether in the whole sample or in two grouped samples; second, both in the overall sample and the grouped sample of state-owned enterprises, job burnout partially mediated the negative relationship between psychological contract breach and job performance of new generation of employees, and past experience of breach positively moderated the negative relationship between psychological contract breach and job performance of new generation of employees; third, in the grouped sample of non-state-owned enterprises, job burnout did not play a significant mediating role in the relationship between psychological contract breach and job performance of new generation of employees, and past breach experience did not play a significant moderating role in this relationship. These findings uncover the psychological mechanism underlying work performance of new generation of employees, and also provide useful theoretical reference for management practices of new generation of employees among different natures of enterprises.

## Introduction

New generation of employees are gradually becoming the main force in China’s labor market (Williams, 2020). However, the practical management experience is still not powerful when facing this new type of employees who have greater interest demands (Wang et al., 2022), more sensitive psychological construction (Zhou and Qian, 2021) and so on. And it is becoming increasing for cracks between new generation of employees and their organizations. Psychological contract breach refers to the perception of employees to feel the organization failure to fulfill the psychological contract. It is becoming more frequent for new generation of employees to have psychological contract breach experience (Samah et al., 2021) and negative working attitude (Yannick and Tim, 2018).

Scholars have recognized the importance of research on new generation of employees management, and have paid more attentions on their turnover tendency (Chen et al., 2021; Liu et al., 2022), innovation behavior (Li et al., 2020; Mou et al., 2021), and job performance (Huang et al., 2015; Liu and Chiu, 2020). But unfortunately, the existing research has primarily focused on the antecedents that positively impact the behavior of new generation of employees, such as sense of values (Huang et al., 2015) and job satisfaction (Zhou and Qian, 2021), and lacked the systematic research on the antecedents that may negatively affect their behaviors, such as psychological contract breach (Yannick and Tim, 2018) and compulsory citizenship behavior (Samah et al., 2021). Meanwhile, more attention has been paid to the direct (Yan et al., 2018) and static (Jayaweera et al., 2021; Samah et al., 2021) mechanism between psychological contract breach and job performance of new generation of employees, and there has few study on the possible indirect and dynamic mechanisms underlying the relationship between such variables, as well as considering the dynamic characteristics of psychological contract formation.

Considering the new generation of employees are more prone to experience psychological contract breach (Kim et al., 2017) which will cause the negative work behavior (Ahn et al., 2021), this study took psychological contract breach as the antecedent variable of new generation of employees’ job performance, and took past breach experience as the moderating variable, so that to explore the mechanism underlying the effect of psychological contract breach on new generation of employees’ job performance from a dual perspective.

As an irreconcilable response for stress (Maslach et al., 2012), job burnout not only damages employees’ physical and mental health (Liu et al., 2022), but also causes negative behaviors (Peng et al., 2019). For example, drawing from social exchange and role theory, Timothy et al. (2021) found the positive correlation between emotional exhaustion and depersonalization, and the negative correlation between job achievement and organizational performance. Based on the job requirements-control model, Liu et al. (2022) proposed that job stress of R&D employees positively predicts their job burnout. Taking the perspective of the job demand-resource theory, Bakker and de Vries (2021) found that job burnout of high-tech workers could reduce their happiness and improve their turnover intention. And studies have explored the direct relationship between job burnout and job performance within a particular occupational group, such as teachers (Peng et al., 2019), nurses (Li et al., 2022), and drivers (Tu et al., 2021), and have failed to consider such issue among more broader groups, such as new generation of employees, and also ignored the antecedent effect of the implicit variable of psychological contract breach on employees’ job burnout.

To explore the relationship between psychological contract breach and job performance of new generation of employees, we combined social exchange theory, fairness theory, and conservation of resource theory, and constructed the framework between psychological contract breach and job performance for new generation of employees with the consideration of the mediating effect of job burnout and the moderating effect of past breach experience. The hypotheses were tested using the overall sample data and grouped data. Our work expands the understanding of the importance of psychological contract breach on job performance of new generation of employees, and also provides a detailed theoretical basis for relevant management practices among different natures of enterprises.

## Literature review and hypotheses

### Psychological contract breach and job performance of new generation of employees

As an implicit agreement between individuals and organizations, a psychological contract reflects employees’ internal beliefs about whether the organization can meet their potential psychological needs (Robinson and Morrison, 2000). When employees feel the organization has failed to fulfill its commitments, psychological contract breach will be generated (Rousseau, 1989). Given that new generation of employees have higher psychological expectations on their organizations, they will have more negative attitudes toward and retaliation behaviors against organizational behavior when they perceive the organization has failed to fulfill its commitments as contract (Samah et al., 2021). The possible theoretical relationship between psychological contract breach and job performance of new generation of employees will be explained in details as follows.

First, according to social exchange theory, there is an exchange relationship between employees and organizations. That is, employees exchange their knowledge, skills, and physical strength to get the pay from organization, so realizing their psychological contracts and promoting continuous reciprocity between them and organizations. However, according to the principle of negative reciprocity, once employees perceive that they are being treated negatively by the organization (that means the organization does not meet its promises), they will respond through negative reciprocal behaviors, such as decreasing trust and loyalty (Yannick and Tim, 2018) and increasing silent psychological behavior (Bari et al., 2020), which will eventually affect their job performances. Second, based on the conservation of resources theory, individuals tend to preserve existing psychological resources to avoid psychological damage. Considering the strong self-awareness and the pursuit of short-term benefits for new generation of employees, the perceived organizational defaults will minimize their work engagement so preventing the loss of their resources (Ma et al., 2019). Third, according to equity theory, employees adjust their work engagement according to the fairness of the perceived relationship between pay and return. If they feel that the pay-return ratio is unfair, their psychological contract will be breached (Subramaniam and Li, 2019). Compared with the non-Cenozoic employees, new generation of employees maybe have more experience of psychological contract breach, because they care more about the fairness of performance issuance procedures and results (Liu and Chiu, 2020). Once the psychological contract is breached, employees will change their input or output to reduce their commitment and effort in exchange for a sense of psychological fairness, which ultimately affects their job performance.

To summarize, by combining social exchange theory, conservation of resource theory, and equity theory, it seems possible if an organization destroys the psychological contract between itself and its new generation of employees, new generation of employees will retaliate by adopting negative reciprocal behaviors, avoiding emotional resources damage, reducing organizational investment and performance. Therefore, hypothesis 1 was proposed as follows:

*H1*: Psychological contract breach has a negative impact on the job performance of new generation of employees. That is, the higher the degree of psychological contract breach, the worse the job performance of new generation of employees will be.

### The mediating role of job burnout

The concept of job burnout can be traced back to Freudenberger (1975) which typically manifests as low mood, demotivation, and loss of motivation for work and life. Subsequently, scholars have expanded the concept of job burnout by different perspectives. For example, Maslach et al. (2012) described job burnout as comprehensive set of symptoms that result from the excessive physical and mental exertion and energy depletion of prolonged work stress, and including three dimensions of emotional exhaustion, work neglect, and low work effectiveness.

Combining resource conservation theory and existential psychology theory, it can be proposed that psychological contract breach has a positive effect on job burnout of new generation of employees. First, according to conservation of resources theory, employees will try to minimize their perceived resource loss by reducing efforts and avoiding work. When employees experience psychological contract breach continuously, their psychological resources become excessively consumed or even exhausted (Xie et al., 2022), which will eventually manifest as job burnout. At the same time, the continuous interruption of psychological resources will increase employees’ frustration at work, which will cause job stress and indirectly lead to job burnout (Wu et al., 2021). Second, according to the existential psychology theory, employees expect to accomplish their attribution needs through their work in an organization. So, when such need could not be met, that means there is a perceived psychological contract breach, a series of complex and integrated emotions (such as disappointment, connivance, and anger) will be elicited, which will further drain their emotional resources. Unlike the non-Cenozoic employees, new generation of employees have higher requirements on salary and working condition (Huang et al., 2015), that is, they have stronger psychological contract. Thus, when they perceive that the organization is not fulfilling its responsibilities and obligations, they are more likely to develop a series of negative work attitudes and behaviors, such as reducing satisfaction, decreasing organizational commitment (Yan et al., 2018) and increasing turnover intention (Timothy et al., 2021).

Moreover, combining the emotional event theory and job requirements-resource theory, it seems that job performance of new generation of employees can be negatively affected by job burnout. First, according to the theory of emotional events, employees’ work attitudes and behavior will be affected by their emotional experiences. As a negative emotional experience, job burnout naturally reduces employee satisfaction (Xie et al., 2022). New generation of employees have unique personalities and psychological characteristics, and rich and diverse job demands (Cafer et al., 2021). Job burnout for a long time can lead to their loss of work motivation (Bakker and de Vries, 2021), thereby impacting their job performance negatively. Second, according to the job requirements and resource theory, job requirements which employees lack or beyond their abilities will induce a loss of energy and resources, and affect performance through job burnout (Maslach et al., 2012). In addition, new generation of employees, who have emotional fragility and poor resistance (Zhou and Qian, 2021), are more likely to be tired of work (Timothy et al., 2021), and have lower work efficiency.

In summary, on the one hand, the psychological contract breach of new generation of employees will induce job burnout (Wang et al., 2021). On the other hand, a persistent state of job burnout for new generation of employees will cause their emotional resources to be continuously depleted and will be difficult to concentrate on work, which will eventually lead to reduced performance. Therefore, according to the above arguments and the principle for proposing the hypothesis about the mediating effect of Wen et al. (2022), hypothesis 2 was proposed as follows:

*H2*: Job burnout mediates the relationship between psychological contract breach and job performance of new generation of employees.

### The moderating role of past breach experience

Employees’ experience of psychological contract breach tends to be accumulated over time (Almer et al., 2019). That is, employees’ psychological contract feelings are susceptible to their past experience of psychological contract breach in work. Employees who have no psychological contract breach experience will be more inclined to trust the organization and thus show higher loyalty to the organization, while employees who experienced psychological contract breach in the past, will be more likely to feel restless and uncertainty (Yannick and Tim, 2018), so will reduce their job engagement (Samah et al., 2021) and increase turnover intention (Chen et al., 2021) to protect themselves. For new generation of employees in China today, they show more obvious characteristics such as utilitarian orientation, innovation orientation, long-term development, internal preference, and interpersonal harmony (Williams, 2020), which will lead them not only care more about economic returns, but also pay more attention on promoting the generation and implementation of creative ideas. Therefore, it is particularly important to consider the dynamics of psychological behavior when researching the relationship between psychological contract breach and job performance of new generation of employees.

Social exchange theory holds that, when employees perceive an unequal psychological contract exchange relationship with their organization, they will adjust their behavior so to back the equilibrium state of exchange relationship again. Employees will reduce performance to cope with the perceived default behavior, which may eventually interfere with the impact of psychological contract breach on their work performance. New generation of employees will adopt actions such as frequent turnover or job-hopping, so to overcome psychological losses and maintain the inner order of their psychology. Employees with previous breach experience show a more prominent tendency toward non-reciprocity than those without previous breach experience (Rousseau, 1989).

According to self-verification theory, people care a lot about what others think of them, and expect others to view them in their own ways, which shows people always care about the dynamic changes of psychological contracts. Once employees perceive that the organization does not commit them, they will deny themselves, and devalue their significance in the organization (Bari et al., 2020). The characteristics of new generation of employees, such as higher requirements for remuneration and stronger self-awareness, lead them be much easier to experience psychological contract breach. If that, they will not concentrate on creating performance for the organization, and their behavioral awareness will also get hurt. In the long run, it will be increased for their awareness of self-protection and will be more likely to have aggressive thoughts or retaliatory behaviors (Samah et al., 2021), which will finally be all reflected on their work performance. Therefore, when experiencing psychological contract breach again, new generation of employees with past breach experience will show stronger and more sensitive behaviors and attitudes than other employees.

Furthermore, the lower an employee’s trust in their organization, the more likely it will be that a psychological contract breach will be perceived (Bari et al., 2020). Trust is largely based on past experiences. Employees can be strongly affected by psychological contract breach experience in a past employment relationship (Robinson and Morrison, 2000). The past experience of psychological contract breach can decrease employees’ trust in their current employer, which will in turn increase the possibility of psychological contract breach for their current employer, and finally reduce their job performance. Based on these assertions, we proposed the following hypothesis:

*H3*: Past psychological contract breach experience positively moderates the relationship between psychological contract breach and job performance of new generation of employees. That is, the more past breach experiences, the stronger the negative impact of psychological contract breach on job performance of new generation of employees will be.

**FIGURE 1:**
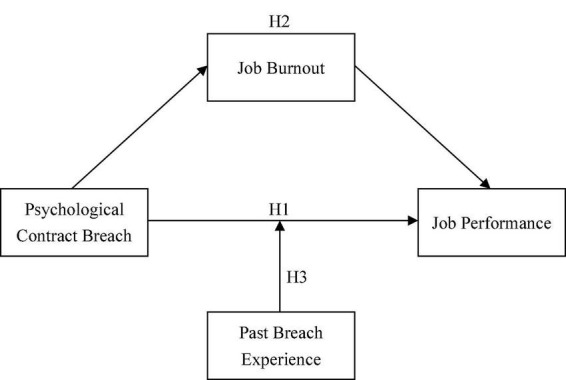
The theoretical model.

To summarize, this study constructed a model of the relationships among psychological contract breach, job burnout, and job performance of new generation of employees (Figure 1).

## Materials and methods

### Measurements of variables

To ensure the reliability and validity of the study, firstly, the mature questionnaire items were revised according to this special research objective. For example, we changed the order of some items, and added reverse questions to check the attitudes of the respondents. Secondly, 56 MBA students in universities of Yunnan Province, who have 2 years or more work experience, were invited to give feedback for the structure and presentation of the revised questionnaire through online and offline methods. Combined with their feedback, some questions were revised again and some less relevant items were deleted, such as the income of the respondents. Later, the revised version of the questionnaire was sent out by random sampling as the pilot. Based on 169 valid questionnaires, the reliability and validity of the questionnaire were tested and all of the test results were within expectations. Finally, the questionnaire used in the pilot was determined as the final version to formal distribution. Besides the moderating and control variables, all other key variables were measured with a five-point Likert scale ranging from 1 (that means the description of certain item is very inconsistent with the perception of the respondent) to 5 (the description of certain item is very consistent with the perception of the respondent), which are shown in Table 1.

**TABLE 1:** Description of variables.

Variable	Number	Measurement items
Psychological contract breach (Psy)	Psy_1_	So far, companies have done very well in fulfilling their commitments to me.
	Psy_2_	So far, companies have fulfilled almost all of the commitments they made to me when they hired me.
	Psy_3_	I think the company has fulfilled its promise to me during my time here.
	Psy_4_	The enterprise did n’t fulfill all the promises in exchange for my contribution.
	Psy_5_	Although I have always kept my promise, the company has repeatedly broken theirs.
Job burnout (Bur)	Bur_1_	Every day I always get up without spirit, facing the work is listless.
	Bur_2_	I feel very tired after working all day.
	Bur_3_	My work often makes me feel bored.
	Bur_4_	I often need to consume too many emotional resources at work.
	Bur_5_	I feel confused about my current career prospects.
	Bur_6_	I have lost enthusiasm for my current job.
	Bur_7_	I think my current job is of little value.
	Bur_8_	I feel I’m not qualified for my current job.
	Bur_9_	Leaders often put me in charge of unimportant things.
	Bur_10_	I often have no confidence in the tasks assigned to me by the leader.
Past breach experience (Pas)	Pas	Have you changed the enterprise? If yes, did your previous enterprise fulfill its promise to you?
Job performance (Per)	Per_1_	I often plan and arrange my work schedule.
	Per_2_	I managed to maintain a high standard of work quality.
	Per_3_	My work can always be completed within the given time.
	Per_4_	My work efficiency is generally high.
	Per_5_	Overall, I can do the tasks required by the company.
	Per_6_	I always want to be assigned a challenging job.
	Per_7_	I often have a pioneering spirit and use my initiative to solve problems in work.
	Per_8_	I often take on extra work to help others or to improve group performance.
	Per_9_	I often work cooperatively with other colleagues in my team.
	Per_10_	When colleagues encounter problems, I give support and encouragement.
	Per_11_	On the whole, I think of my company and help other colleagues voluntarily.

### Explanatory variable

The explanatory variable in this study was psychological contract breach (denoted by Psy), which was measured using Rousseau’s (1989) 5-item scale. An example item is, “So far, enterprises have done very well in fulfilling their commitments to me.”

### Explained variable

The explained variable in this study was job performance of new generation of employees (denoted by Per). Using the instrument developed by Borman and Motowidlo (1993), we measured job performance of new generation of employees using 11 items, such as “I often plan and arrange my work schedule.”

### Intermediary variable

The intermediary variable in this study was job burnout (denoted by Bur), which was assessed using items from Li and Shi (2003) and Maslach et al. (2012). The scale is more in line with China’s situation. Job burnout was assessed using 10 items, such as “Every day I always get up without spirit, facing the work is listless.”

### Moderating variable

The moderating variable of this study was past breach experience. Referring mainly to Robinson and Morrison (2000), we measured past breach experience by asking participants, “Have you changed enterprises? If yes, did your previous enterprise fulfill its promise to you?” (1 = no replacement, 2 = never default, 3 = basic compliance, 4 = one violation, and 5 = two or more violations).

### Control variables

Based on the research work of Marchand et al. (2018), Samina et al. (2018), Wendy et al. (2018) and Lu et al. (2021), this study selects five control variables, which are age, sex, working years, nature of enterprise, and level of education. The measurements for those control variables are as follows: for age, 1 = 20–25 years old, 2 = 26–30 years old, 3 = 31–35 years old, 4 = 36–40 years old, 5 = 41 years old and above; for sex, 1 = male, 2 = female; for working years, 1 = less than 2 years, 2 = 2–5 years, 3 = 6–8 years, 4 = more than 8 years; for nature of enterprise, 1 = state-owned enterprises, 2 = non-state-owned enterprises; for level of education, 1 = college degree and below, 2 = undergraduate degree, 3 = Master degree and above.

### Description of the sample

Since the variables in this study were not obtained from public information, a questionnaire was used to collect data. This study selected Yunnan Province as the test area based on the following considerations. First, the main team members of this study are living or working in Yunnan Province, which gives the convenience of questionnaire distribution and collection. Second, according to the data of the seventh national census, the total population aged 20–30 in Yunnan Province accounts for 23.675% of the over whole working-age population in Yunnan province. That means, new generation of employees accounted for a quarter of the total population of working-age in Yunnan Province, which explains the representativeness of samples in this study. Third, compared with other provinces in China, Yunnan Province is relatively undeveloped in education level and economic situation. Taking new generation of employees in such area of China as sample, it will benefit to exploring the overall psychological contract status of new generation of employees in China.

Then, considering the different contributions and importance of different industries, five industries, that are transportation, tourism, education, medical services, and computer science in Yunnan Province, were selected as the specific subjects. This is mainly based on the following considerations: first, the income of tourism and transportation industry account for more than 60% of GDP in Yunnan province, which means its importance for the economy of Yunnan Province; second, although it is only about 5% for the contribution of the computer science industry to GDP in Yunnan province, it was still included as the subject because the high ratio of new generation of employees in such industry; third, considering the importance of education expenditure and medical expenditure to people’s livelihood, they were also taken as the sample industries.

Subsequently, considering the convenience and representativeness of data collection, this paper selected ten enterprises whose proportion of new generation of employees exceeded 30% from these five industries as the sample enterprises. Among them, three from the transportation industry, two from the tourism industry, two from the education industry, two from the medical services industry, and one from the computer sciences industry. And five enterprises were state-owned and other five were non-state-owned.

The research team distributed a total of 368 online questionnaires to the 10 sample enterprises from January to March 2021, and recovered 368. After removing 133 questionnaires that did not conform to the age group of new generation of employees (20–30 years), had incomplete answers, and had inconsistent answers to the reverse questions, 235 vivid questionnaires were obtained, and the efficiency rate is 63.859%.

Table 2 shows the descriptive statistics of the sample. In terms of sex, male subjects accounted for 56.170% and female subjects accounted for 43.830% of the sample; over 60% of subjects were 20–25 years old; holders of a college degree and below accounted for 60.851%, holders of an undergraduate degree accounted for 37.021%, and holders of a Master’s degree and above accounted for 2.128%; 57.872% of samples worked for state-owned enterprises, and 42.128% for non-state-owned enterprises. Participants with less than 2 years working experience accounted for 57.872%, those with 2–5 years of working experience accounted for 27.660%, those with 5–8 years of working experience accounted for 11.106%, and those with more than 8 years of working experience accounted for 3.404%.

**TABLE 2:** Descriptive statistics for the sample.

Characteristic	Classification	Frequency	Percentage	Characteristic	Classification	Frequency	Percentage
Sex	Male	132	56.170	Education	College degree and below	143	60.851
	Female	103	43.830		undergraduate college	87	37.021
Age	20–25 years old	144	61.277		Postgraduate and above	5	2.128
	26–30 years old	91	38.723	Length	Less than 2 years	136	57.872
Nature	State-owned enterprise	136	57.872		2–5 years	65	27.660
	Non-state-owned enterprises	99	42.128		5–8 years	26	11.106
					More than 8 years	8	3.404

### Testing for reliability and validity

The Cronbach’s α coefficients for psychological contract breach, job burnout, and new generation of employees’ job performance were 0.858, 0.917, and 0.919, respectively. These results indicate that all the items in this study had good internal consistency and reliability.

An exploratory factor analysis was conducted using the Kaiser–Meyer–Olkin test and Bartlett’s test of sphericity. The Kaiser–Meyer–Olkin values for psychological contract breach, job burnout, and job performance were 0.793, 0.932, and 0.915, respectively, and passed Bartlett’s spherical test (*p* = 0.000 < 0.01), which indicated that the construct validity of the questionnaire was good.

Average variance extracted values and combined reliability measurements were made to gauge the convergent validity of the sample. The results showed that the average variance extracted values of psychological contract breach, job burnout, and job performance were 0.525, 0.535, and 0.510, respectively (all greater than the threshold value of 0.5), and the corresponding combined reliability values were 0.846, 0.918, and 0.919, respectively (all greater than the threshold value of 0.7), which indicated that the questionnaire had high aggregation validity.

The correlations between variables were calculated using the Pearson correlation coefficient matrix. As shown in Table 3, psychological contract breach was significantly negatively correlated with job performance of new generation of employees (*r* = –0.486, *p* < 0.01), psychological contract breach was significantly positively correlated with job burnout (*r* = 0.544, *p* < 0.01), job burnout was significantly negatively correlated with job performance of new generation of employees (*r* = –0.470, *p* < 0.01), and the interaction term of psychological contract breach and past breach experience was significantly negatively correlated with job performance of new generation of employees (*r* = –0.327, *p* < 0.01). Therefore, all the above-mentioned hypotheses have been preliminarily verified.

**TABLE 3:** Correlation analysis of the variables.

	Gender	Education	Age	Nature	Length	Psy	Bur	Psy* Pas	Per
Sex	1								
Education	0.136*	1							
Age	0.213**	0.269**	1						
Nature	0.272**	−0.171**	0.092	1					
Length	0.233**	0.154*	0.443**	−0.014	1				
Psy	0.083	0.283**	0.167*	−0.025	0.239**	1			
Bur	0.147*	0.166*	0.092	0.003	0.283**	0.544**	1		
Psy* Pas	0.108	0.223**	0.279**	0.200**	0.099	0.532**	0.234**	1	
Per	−0.149*	−0.183**	−0.059	−0.016	−0.178**	−0.486**	−0.470**	−0.327**	1

*Significant at *p* < 0.1; **Significant at *p* < 0.05; the constant terms are omitted.

## Results

### Testing of hypotheses

We first tested the relationship between psychological contract breach and job performance of new generation of employees. First, by taking the five control variables (sex, education, age, enterprise kind, and work experience) as independent variables, and job performance of new generation of employees as the dependent variable, Model 1 was constructed. Model 2, as shown in Table 4, was then built by adding psychological contract breach as an independent variable in Model 1. After controlling for the control variables, the negative impact of psychological contract breach on job performance of new generation of employees was significant (β = −0.344, *p* < 0.01), thus supporting H1.

**TABLE 4:** Results of relationship between psychological contract breach and job performance of new generation of employees (*N* = 235).

Variable	Model 1 Per	Model 2 Per	Model 3 Bur	Model 4 Per	Model 5 Per
Constant	4.411**	4.920**	0.700**	5.068**	4.093**
Sex	−0.115	−0.118	0.121	−0.093	−0.110
Education	−0.189*	−0.060	0.014	−0.057	−0.041
Age	0.102	0.108	−0.148	0.077	0.100
Nature	−0.016	−0.011	0.006	−0.009	−0.008
Length	−0.120*	−0.055	0.172**	−0.019	−0.060
Psy		−0.344**	0.491**	−0.240**	−0.333**
Bur				−0.211**	
Pas					−0.001
Psy* Pas					−0.093**
*R* ^2^	0.071	0.257	0.333	0.307	0.281
Adj-*R*^2^	0.051	0.238	0.315	0.286	0.255
*F*	3.496**	13.153**	18.964**	14.363**	11.022**

*Significant at *p* < 0.1; **Significant at *p* < 0.05.

To test the mediating effect of job burnout in this relationship, Model 3 which was shown in Table 4 was constructed by taking psychological contract breach as the independent variable and job burnout as the dependent variable. After controlling for sex, education, age, enterprise kind, and working years, psychological contract breach had a significant positive impact on job burnout (β = 0.491, *p* < 0.01). Then, taking both psychological contract breach and job burnout as independent variables, and job performance of new generation of employees as the dependent variable, Model 4 was constructed and shown in Table 4. The regression results show that both psychological contract breach and job burnout had a significantly negative impact on job performance of new generation of employees (β = –0.240 and –0.211, respectively, and both *p* < 0.01). So that, job burnout played a partial mediating role in the relationship between psychological contract breach and job performance of new generation of employees, thereby verifying H2.

To test the moderating effect of past breach experience, the natural term of past breach experience, and its interaction term with past breach experience were added to Model 2, then Model 5 was constructed in Table 4. After controlling for sex, educational background, age, enterprise kind, and working years, the regression coefficients of psychological contract breach and its interaction with past breach experience were significantly negative (β = −0.333 and −0.093, respectively, and both *p* < 0.01). This shows that past breach experience augmented the negative relationship between psychological contract breach and job performance of new generation of employees; that is, past breach experience had positively moderated the relationship between psychological contract breach and job performance of new generation of employees, thus verifying H3.

**FIGURE 2:**
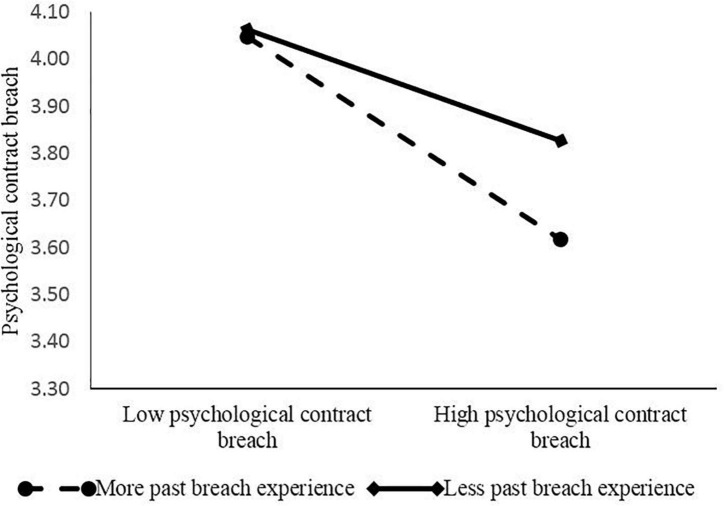
Interaction between past breach experience and psychological contract breach.

In order to analyze the moderating effect of past breach experience in detail, this study takes the average value of past breach experience plus or minus one standard deviation as the grouping standard, and then characterizes the relationship between psychological contract breach and job performance under such different levels of past breach experience. As shown in Figure 2, compared with the low level of past breach experience, when new generation of employees have a high level of past breach experience (as shown by the dotted line in Figure 2), the negative effect of psychological breach on their job performance was more obvious, and the corresponding curve slope is larger.

To further explore the relationship between psychological contract breach and job performance of new generation of employees, we grouped participants according to the nature of enterprise they worked for. And we found that the relationship between psychological contract breach and their job performance was different among different group samples. The specific grouping regression results are shown in Table 5.

**TABLE 5:** Test results of the relationship between psychological contract breach and job performance of new generation of employees (grouped by the nature of enterprises).

Variable	State-owned enterprise group (*N* = 136)					Non-state-owned enterprise group (*N* = 99)				
	Model 6 Per	Model 7 Per	Model 8 Bur	Model 9 Per	Model 10 Per	Model 11 Per	Model 12 Per	Model 13 Bur	Model 14 Per	Model 15 Per
Constant	4.472**	4.916**	0.446*	5.039**	4.112**	4.113**	4.755**	1.211**	4.924**	3.853**
Sex	−0.144	−0.131	0.321**	−0.043	−0.108	−0.173	−0.163	−0.053	−0.171	−0.170
Education	−0.125	0.040	−0.089	0.015	0.061	−0.127	−0.077	0.015	−0.074	−0.036
Age	0.054	0.093	−0.162	0.048	0.031	0.112	0.099	−0.108	0.084	0.164
Length	−0.187**	−0.143*	0.195**	−0.090	−0.135*	0.070	0.136	0.086	0.148	0.112
Psy		−0.342**	0.556**	−0.189*	−0.334**		−0.356**	0.417**	−0.298**	−0.336**
Bur				−0.275**					−0.139	
Pas					0.099					−0.083
Psy* Pas					−0.140**					−0.081
*R* ^2^	0.133	0.294	0.469	0.360	0.341	0.049	0.276	0.199	0.303	0.325
Adj-*R*^2^	0.106	0.267	0.448	0.330	0.304	0.008	0.237	0.156	0.257	0.273
*F*	5.002**	10.828**	22.952**	12.068**	9.441**	1.205	7.077**	4.625**	6.656**	6.252**

*Significant at *p* < 0.1; **Significant at *p* < 0.05.

In the group of state-owned enterprises, by comparing Model 6 and Model 7, we found that when variables such as sex, education, age, and working years were controlled, the negative effect of psychological contract breach on job performance was significant (β = −0.342, *p* < 0.01), thereby validating H1. Combined with Model 6, Model 8, and Model 9, job burnout partially mediated the relationship between psychological contract breach and job performance of new generation of employees, thereby validating H2. Combined with Model 7 and Model 10, past breach experience had a significant positive moderating effect on the relationship between psychological contract breach and job performance of new generation of employees, thereby validating H3.

However, in the group of non-state-owned enterprises, compared with Model 11 and Model 12, when controlling for sex, education, age, working years, and other variables, psychological contract breach also showed a significant negative impact on job performance of new generation of employees (β = −0.356, *p* < 0.01), thereby also validating H1. Combining Model 12, Model 13, and Model 14, we found that job burnout did not mediate the relationship between psychological contract breach and job performance of new generation of employees, so H2 was not supported. Combining Model 12 and Model 15, we found that the moderating effect of past breach experience on the relationship between psychological contract breach and job performance of new generation of employees was also not significant, so H3 was not supported.

## Conclusion and discussion

### Conclusion

Based on the social exchange theory and conservation of resource theory, this study starts research on the relationship between psychological contract breach and job performance of new generation of employees from the dual perspectives of job burnout and past breach experience. Using data from 235 participants, the proposed hypotheses were verified in both overall and grouped samples, and the following conclusions were obtained. First, both in the overall sample and grouped samples, both job burnout and psychological contract breach had a significant negative impact on job performance of new generation of employees. Second, in the overall sample and within the state-owned enterprise group, job burnout partially mediated the relationship between psychological contract breach and job performance of new generation of employees, and past breach experience also played a significant positive moderating role in this relationship. Third, in the non-state-owned enterprise group, both job burnout and past breach experience did not significantly affect the relationship between psychological contract breach and job performance of new generation of employees.

Job burnout did not play a mediating role in the relationship between psychological contract breach and job performance in employees of non-state-owned enterprises. The possible explanations are as follows. First, compared with state-owned enterprises, non-state-owned enterprises are more diversified, more challenging, and more autonomous in their job characteristics and organizational culture. These features are more compatible with the work values of Chinese new generation of employees, so that new generation might be less prone to job burnout. Specifically, compared with older-generation employees, new generation of employees place more emphasis on self-feeling and experience (Huang et al., 2015), are willing to accept more diversified, interesting, challenging, and competent work (Li et al., 2020), which matches the work characteristics of non-state-owned enterprises (Ma et al., 2019) and organizational culture well (Huang et al., 2015). Second, the ability-based promotion incentive system in non-state-owned enterprises may be more conducive to realize the self-actualization of new generation of employees, thereby reducing their job burnout. Unlike state-owned enterprises that regard qualifications and GUANXI as the main factors for promotion, non-state-owned enterprises pay more attention on individual capability, and adopt a fairer and more impartial system to assess promotion possibilities. This not only strengthens the self-realization consciousness of new generation of employees (Huang et al., 2015), but also stimulates their willingness to work (Timothy et al., 2021). This could also explain our findings that the average job burnout was higher among employees of state-owned enterprises than the one among employees of non-state-owned enterprises.

Past breach experience failed to play a moderating role in the relationship between psychological contract breach and job performance of new generation of employees in non-state-owned enterprises. The possible explanations are as follows. First, compared with state-owned enterprises, non-state-owned enterprises have more differentiated salary system which may inhibit the negative impact of psychological contract breach on job performance. In general, non-state-owned enterprises have formed a performance-based salary incentive system (Zhang, 2021). That is, the salary of employees is based on their performance, which can effectively inhibit the negative impact of psychological contract breach on their job performance to some extent (Kim et al., 2017). Second, the more flexible employment climate in non-state-owned enterprises might limit the accumulation of the negative effect of past breach experience on job performance. Among non-state enterprises, the psychological contract breach of new generation of employees significantly impacts their turnover intention (Wang et al., 2021). Therefore, when they perceive organizational betrayal repeatedly, they will be more likely to choose to leave, so there is little accumulation of their psychological contract breach experience within the same enterprise. In contrast, influenced by the idea of “Iron Rice Bowl (a lifelong secure job or position),” employees of state-owned enterprises are highly dependent on the organization itself (Lu et al., 2021). Even the experience of psychological contract breach continues to increase, they will still not choose to leave, which may instead result in job burnout or a drop in job performance. Third, more targeted incentives for non-state-owned enterprises may effectively reduce the possibility of psychological contract breach for new generation of employees. Normally, non-state-owned enterprises respond to and recognize employees’ contribution and work performance through various incentives in time (Zhang, 2021), and some high-tech enterprises have even set up incentive mechanisms for new generation of knowledge workers (Fan et al., 2021). By contrast, state-owned enterprises focus more on executive’s pay incentives and promotion incentives (Duan et al., 2021), with few incentives for the first line employees and new generation of employees. Although some state-owned enterprises have implemented ESOP (shorted for Employee Stock Ownership Plans) in mixed-ownership reform, its implementation effect is still far from that of private enterprises (Ma and Zhang, 2021).

### Theoretical implications

Our findings have several theoretical implications. First, previous studies have focused on the negative employee behaviors caused by psychological contract breach (Ma et al., 2019; Samah et al., 2021), and missed the specificity of employees and the difference of past breach experience. Therefore, considering the particularity of new generation of employees in terms of demands (Huang et al., 2015) and psychological construction (Fan et al., 2021), this study actively explored the mechanism underlying the effect of psychological contract breach on work performance of new generation of employees. Moreover, considering that the continuity of past breach experience might impact employee behavior, this study also examined the moderating effect of past breach experience of new generation of employees to expand the research perspective.

Second, the effect of job burnout on the relationship between psychological contract breach and job performance of new generation of employees has not been systematically studied. And the existing literature on the relationship between job burnout and employee job performance has primarily been concerned with specific enterprises in a certain industry such as high-tech new energy companies (Bakker and de Vries, 2021), or specific groups such as science and technology workers (Liu et al., 2022). Moreover, previous work has failed to consider the different relationships among different kinds of enterprises (Timothy et al., 2021). Unlike most existing studies, this study examined how the effect of job burnout on the relationship between psychological contract breach and job performance differs between employees of state-owned and non-state-owned enterprises. So the conclusions should be more targeted according to the kind of enterprises.

### Managerial implications

Our findings have several practical implications. First, our results contribute to building the maintenance mechanisms of psychological contract for new generation of employees. The empirical results of the overall and grouped samples show that psychological contract breach in new generation of employees directly reduces their job performance and induces the indirect impact of job burnout on job performance. Therefore, building a psychological contract maintenance mechanism is particularly important for an organization’s development. In doing so, organization should integrate the unique work values of new generation of employees. Namely, it should provide work support, interpersonal support, and psychological support to help new generation of employees integrate into the organization as soon as possible. Moreover, to fulfill the commitments made to new generation of employees, it should know their psychological status and changes through various channels, and take remedial measures for the unfulfilled commitments in time.

Second, attention should be paid to the mitigation effect of job burnout. Based on the empirical results of the overall sample and state-owned enterprise employees, it seems clear that job burnout plays a significant partial mediating role in the relationship between psychological contract breach and job performance of new generation of employees. Considering that psychological contract breach cannot be completely eliminated in the organization, enterprises (especially state-owned enterprises) should design a more incentive-driven salary system, more objective and fair performance evaluation system, more rich training system and promotion channels to improve the job performance of new generation of employees. These measures could help to alleviate job burnout of new generation of employees in multiple ways.

Third, it should establish the identification mechanism of psychological contract breach experience for new generation of employees. It was found that past breach experience intensifies the negative impact of psychological contract breach on the job performance of new generation of employees. Therefore, state-owned enterprises should pay more attention on individuals’ past breach experience and its causes in talent recruitment and selection so to avoid the accumulation of psychological contract breach experience among employees.

### Limitations and future research

Two limitations of this study should be mentioned. First, to avoid the complexity of comparative studies in different regions, this study selected new generation of employees in Yunnan province only. Therefore, the generalizability of our findings needs to be further explored. Second, this study mainly adopted the online questionnaire survey due to the COVID-19 pandemic, so there is still room for improvement in terms of data collection.

## Data availability statement

The raw data supporting the conclusions of this article will be made available by the authors, without undue reservation.

## Ethics statement

Ethical review and approval was not required for the study on human participants in accordance with the local legislation and institutional requirements. Written informed consent for participation was not required for this study in accordance with the national legislation and the institutional requirements.

## Author contributions

DY contributed to establishment of the theory, revision of the manuscript, and quality inspection of the manuscript. KY and XZ contributed to data collection, data analysis, and the manuscripts written. YL, SW, MD’A, and GR contributed to the framework establishment. All authors contributed to the article and approved the submitted version.
